# Main Functions and Taxonomic Distribution of Virulence Genes in *Brucella melitensis* 16 M

**DOI:** 10.1371/journal.pone.0100349

**Published:** 2014-06-25

**Authors:** Aniel Jessica Leticia Brambila-Tapia, Dagoberto Armenta-Medina, Nancy Rivera-Gomez, Ernesto Perez-Rueda

**Affiliations:** Departamento de Ingeniería Celular y Biocatálisis, Instituto de Biotecnología, Universidad Nacional Autónoma de México, Cuernavaca, Morelos, México; Institut National de la Recherche Agronomique, France

## Abstract

Many virulence genes have been detected in attenuated mutants of *Brucella melitensis* 16 M; nevertheless, a complete report of these genes, including the main Cluster of Orthologous Groups (COG) represented as well as the taxonomical distribution among all complete bacterial and archaeal genomes, has not been analyzed. In this work a total of 160 virulence genes that have been reported in attenuated mutants in *B. melitensis* were included and analyzed. Additionally, we obtained 250 *B. melitensis* randomly selected genes as a reference group for the taxonomical comparisons. The COGs and the taxonomical distribution profile for 789 nonredundant bacterial and archaeal genomes were obtained and compared with the whole-genome COG distribution and with the 250 randomly selected genes, respectively. The main COGs associated with virulence genes corresponded to the following: intracellular trafficking, secretion and vesicular transport (U); cell motility (N); nucleotide transport and metabolism (F); transcription (K); and cell wall/membrane/envelope biogenesis (M). In addition, we found that virulence genes presented a higher proportion of orthologs in the *Euryarchaeota* and *Proteobacteria* phyla, with a significant decrease in *Chlamydiae*, *Bacteroidetes*, *Tenericutes*, *Firmicutes* and *Thermotogae*. In conclusion, we found that genes related to specific functions are more relevant to *B. melitensis* virulence, with the COG U the most significant. Additionally, the taxonomical distribution of virulence genes highlights the importance of these genes in the related *Proteobacteria*, being less relevant in distant groups of organisms with the exception of *Euryarchaeota*.

## Introduction

Bacteria of the genus *Brucella* are the etiological agents of brucellosis, the most widely spread zoonotic disease worldwide. *Brucella* spp. are able to infect a wide range of mammals, including humans, with an estimated 500,000 new reported human cases per year [1], [2]. Although bacteria belonging to this genus, such as *B. melitensis*, *B. suis*, *B. abortus* and *B. canis*, are known to infect humans, only *B. melitensis* and *B. abortus* are considered the main causal agents of human infections [3], [4].

*B. melitensis* is a Gram-negative facultative intracellular pathogen that belongs to the alpha-2 proteobacteria group. This bacterium has the ability to infect phagocytic macrophages and nonphagocytic cells (epithelial cells, fibroblasts and osteoblasts), and its virulence relies on the ability to replicate inside these cells [5], [6]. *Brucella* lacks known bacterial pathogenic factors that can directly harm eukaryotic cells, such as cytolisins, exotoxins, exoproteases and exoenzymes, nor does it express pathogenic determinants, like fimbriae, capsules, antigenic variation and plasmids [5], [6]. All these data suggest that the bacterium produces direct tissue damage, probably by the activation of host immune responses [5]. Despite the absence of classical virulence factors, many virulence genes have been detected via random [7], [8] and directed [9] mutagenesis in attenuated phenotypes. The best studied are the *virB* operon, which encodes the Type IV secretion system (T4SS), and genes related to lipopolysaccharide production and membrane proteins [10], [11], [12]. To date, an integrative analysis of these genes in *B. melitensis* has not been addressed; therefore, the main goal of this work is to achieve a comprehensive comparison of virulence-related genes identified in *B. melitensis* 16 M under different approaches, to gain insights into their main functions and their taxonomical distribution across bacterial and archaeal genomes.

## Material and Methods

In this analysis, we included all the identifiable genes (by the BME locus) reported to date *in vivo* or in cells with an attenuated phenotype in *B. melitensis* 16 M [7]–[10], [12]–[24]. We achieved a comparison of the functional annotations based on the Cluster of Orthologous Groups (COG) classification in relation to the whole-genome COG distribution. Additionally, a taxonomical distribution analysis profile of these genes was conducted and the results were compared with randomly selected genes of *B. melitensis*.

### Functional annota tions

The functional annotations were retrieved from the DOE Joint Genome Institute Integrated Microbial Genomics Project server (http://img.gji.doe.gov).

### Identification of orthologous genes

The orthologs for each gene in 789 nonredundant bacterial and archaeal genomes that have been completely sequenced were obtained by using the bidirectional best hits (BDBHs) definition in the protein sequence through depurated genomes at 95% identity, with an E-value of ≤1e-6, as described elsewhere [25]. The taxonomical distribution of the virulence genes was compared with the taxonomical distribution of 250 randomly selected genes of the *B. melitensis* 16 M genome that did not overlap with virulence genes; these randomly selected genes were obtained using the function of random gene selection of the RSA tools database (http://rsat.ulb.ac.be). These genes are described in Supplementary Table S1.

### Statistical analysis

The descriptive statistics for qualitative variables consisted of frequencies and percentages and for quantitative variables they consisted of medians and ranges. For the comparison of the COG classification and the taxonomical distribution, the chi-squared test and Fisher exact test were performed. For the comparison of the number of orthologs between virulence and randomly selected genes, we used the Mann-Whitney U test (considering the nonparametric distribution of the data). The statistical analysis was carried out with the software SPSS v 10.0 and Epi-info. Statistical significance was set at p≤0.05.

## Results

A total of 160 virulence genes of *B. melitensis* 16 M identified in attenuated mutants were gathered from the literature. The BME locus, functional description, COG annotation and number of orthologs identified in all the bacterial and archaeal genomes included, as well as the reference, are listed in Table 1.

**Table 1:** Virulence genes in *B. melitensis* 16 M reported in the literature.

Gene id	Gene name	Gene function	COG	N.O.	Reference
BMEI0898	BMEI0898	Acyl-CoA transferase	C	104	[7]
BMEI0972	*gor*	Glutathione reductase	C	0	[8]
BMEI1749	*glpD*	Glycerol-3-phosphate dehydrogenase	C	173	[8]
BMEII0378	*fdhA*	Formate dehydrogenase alpha chain	C	209	[7]
BMEII0429	BMEII0429	Glycerol-3-phosphate dehydrogenase	C	271	[8]
BMEII0759	BMEII0759	Cytochrome D ubiquinol oxidase subunit II	C	419	[8]
BMEII0760	BMEII0760	Cytochrome D ubiquinol oxidase subunit I	C	449	[8]
BMEII0823	*glcK*	Glycerol kinase, partial	C	472	[7]
BMEII1001	*norE*	Heme-copper oxidase subunit III	C	52	[7]
BMEII0761	*cydC*	Transport ATP-binding protein	C/O	190	[8]
BMEII0762	*cydD*	Transport ATP-binding protein	C/O	222	[8]
BMEI0025	BMEI0025	L-sorbose dehydrogenase (FAD)	E	155	[8]
BMEI0433	*dppA*	Periplasmic dipeptide transport protein, partial	E	171	[7]
BMEI0933	*cysK*	Cysteine synthase A	E	212	[17]
BMEI1759	*metH*	B12-dependent methionine synthase	E	510	[17]
BMEII0040	*gltB*	Glutamate synthase [NADPH] large chain	E	566	[8]
BMEII0136	*pheB*	Homoprotocatechuate 2,3-dioxygenase	E	81	[7]
BMEII0626	BMEII0626	Membrane dipeptidase	E	269	[7]
BMEI1301	*dapA*	Dihydrodipicolinate synthase	E/M	728	[7]
BMEII0285	*dppB*	Peptide ABC transporter permease	E/P	105	[8]
BMEI1606	*prlS*	Sensory transduction histidine kinase	E/R	185	[17], [18]
BMEI0233	*purH*	Bifunctional phosphoribosylaminoimidazolecarboxamide formyltransferase/IMP cyclohydrolase	F	665	[8]
BMEI0295	*purK*	Phosphoribosylaminoimidazole carboxylase atpase subunit	F	566	[8]
BMEI0296	*purE*	Phosphoribosylaminoimidazole carboxylase catalytic subunit	F	706	[8], [13]
BMEI1090	BMEI1090	Deoxyguanosinetriphosphate triphosphohydrolase-like protein	F	464	[20]
BMEI1123	*purS*	Phosphoribosylformylglycinamidine synthase subunit	F	336	[8]
BMEI1124	*purQ*	Phosphoribosylformylglycinamidine synthase I	F	694	[8]
BMEI1127	*purL*	Phosphoribosylformylglycinamidine synthase II	F	700	[8]
BMEI1240	*purM*	Phosphoribosylaminoimidazole synthetase	F	685	[8]
BMEI1488	BMEI1488	Amidophosphoribosyltransferase	F	688	[8]
BMEI1519	*purD*	Phosphoribosylamine-glycine ligase	F	706	[8]
BMEI1204	*ppx*	Exopolyphosphatase	F/P	216	[8], [24]
BMEI1396	*pmm*	Phosphomannomutase	G	5	[7]
BMEI1886	BMEI1886	Phosphoglucomutase	G	304	[8]
BMEI2031	*ptsH*	Phosphocarrier protein HPr	G	273	[8]
BMEII0485	*galcD*	D-galactarate dehydratase	G	185	[7]
BMEII0591	BMEII0591	Sugar transport system permease	G	119	[7]
BMEII0624	*ugpA*	SN-glycerol-3-phosphate transport system permease	G	136	[7]
BMEII0899	BMEII0899	Phosphomannomutase	G	32	[8]
BMEII0935	BMEII0935	Nickel resistance protein	G	184	[7]
BMEII1045	BMEII1045	HAD superfamily protein involved in N-acetyl-glucosamine catabolism	G	99	[7]
BMEII1095	*sbgE*	Aldolase	G	130	[8]
BMEI1415	*rfbD*	O-antigen export system permease protein	G/M	226	[8]
BMEI1416	*rfbE*	O-antigen export system ATP-binding protein RFBB	G/M	388	[8]
BMEI1427	*capD*	Capsular polysaccharide biosynthesis protein	G/M	390	[8]
BMEI0657	*btuB*	Metal chelate outer membrane receptor	H	290	[8]
BMEI1902	BMEI1902	Molybdopterin biosynthesis protein	H	97	[17]
BMEI0705	*cobB*	Cobyrinic acid A,C-diamide synthase	H	374	[7], [17]
BMEI0624	*ilvC*	Keto-acid reductoisomerase	H/E	633	[7]
BMEI1553	BMEI1553	Transporter	I	33	[8]
BMEI0983	*rluA*	Ribosomal large subunit pseudouridine synthase C	J	313	[8]
BMEI1057	*cafA*	Ribonucleases E/zinc metalloprotease	J	455	[8]
BMEI1775	*rph*	Ribonuclease PH	J	482	[8]
BMEI0357	BMEI0357	AsnC family transcriptional regulator	K	64	[7]
BMEI0371	*virF*	Regulatory factor (rpoE)	K	430	[8]
BMEI0378	*rpoH2*	RNA polymerase factor sigma 32	K	320	[14]
BMEI0430	*nolR*	Nodulation protein NOLR	K	124	[16]
BMEI0508	*greA*	Transcription elongation factor	K	651	[8]
BMEI0513	*lysR*	LysR family transcriptional regulator	K	86	[8]
BMEI0781	*rpoA*	DNA-directed RNA polymerase subunit alpha	K	700	[7]
BMEI0808	*merR*	MerR family transcriptional regulator	K	121	[8]
BMEI1178	*merR*	MerR family transcriptional regulator	K	181	[8]
BMEI1297	*rpoZ*	DNA-directed RNA polymerase subunit omega	K	256	[8]
BMEI1364	*mucR*	Transcriptional regulator protein	K	81	[8]
BMEI1776	*hrcA*	Heat-inducible transcription repressor	K	462	[8]
BMEII0116	*gntR10*	Gntr family transcriptional regulator	K	218	[16]
BMEII1066	BMEII1066	Pyruvate dehydrogenase complex repressor	K	209	[7]
BMEII1116	*vjbR*	Transcriptional activator LuxR familily	K	23	[8], [21]
BMEI0040	*xerD*	Site-specific tyrosine recombinase	L	609	[8]
BMEI0334	*ruvB*	Holliday junction DNA helicase	L	688	[8]
BMEI1307	*xerC*	Integrase	L	94	[8]
BMEI2023	*uvrD/rep*	ATP-dependent nuclease subunit a	L	371	[8], [24]
BMEII0527	*xseA*	Exodeoxyribonuclease VII large subunit	L	612	[7]
BMEI0275	*mgpS*	ATP-dependent DNA helicase	L/K/J	78	[17]
BMEI0271	*mgtA*	Monofunctional biosynthetic peptidoglycan transglycosylase	M	209	[17]
BMEI0359	*macA*	Periplasmic protein of efflux system	M	327	[7]
BMEI0921	*galE*	UDP-glucose 4 epimerase	M	383	[20]
BMEI0997	*wbdA*	Mannosyltransferase	M	5	[8], [12]
BMEI0998	*wboA*	Glycosyltransferase	M	1	[22]
BMEI1249	*omp25*	25 kda outer-membrane immunogenic protein precursor	M	14	[10]
BMEI1302	*mltE*	Soluble lytic murein transglycosylase	M	166	[8]
BMEI1326	BMEI1326	Hypothetical protein	M	12	[8], [24]
BMEI1393	BMEI1393	Mannosyltransferase	M	221	[8]
BMEI1413	*gmd*	GDP-mannose 4,6-dehydratase	M	341	[8]
BMEI1414	*perA*	Perosamine synthetase	M	385	[8]
BMEI1426	BMEI1426	Undecaprenyl-phosphate alpha-N acetylglucosaminyltransferase	M	89	[8]
BMEII0260	*lepA*	GTP-binding protein	M	698	[8]
BMEII0380	*acrA*	Acriflavin resistance protein A	M	148	[8]
BMEII0472	*mtrC*	Membrane fusion protein	M	89	[8]
BMEII0150	*fliC*	Flagellin	N	135	[9]
BMEII0154	*motB*	Flagellar motor protein	N	176	[9]
BMEII0159	*flgE*	Flagellar hook protein	N	285	[9]
BMEII0161	*flgL*	Flagellar hook-associated protein	N	30	[9]
BMEII1107	*flgF*	Flagellar basal-body rod protein, partial	N	32	[24]
BMEII0035	*virB11*	ATPase VIRB11-like protein	N/U	419	[8]
BMEII0151	*fliF*	Flagellar M-ring protein, partial	N/U	340	[7], [9]
BMEII0152	*fliF2*	Flagellar M-ring protein	N/U	134	[9]
BMEII0166	*flhA1*	Flagellar biosynthetic protein	N/U	367	[9]
BMEII0167	*flhA2*	Flagellar, biosynthesis protein partial	N/U	372	[9]
BMEI0193	BMEI0193	Hypothetical protein	NC	1	[8]
BMEI0540	BMEI0540	Hypothetical protein	NC	1	[8]
BMEI1229	BMEI1229	Exonuclease	NC	7	[7]
BMEI1339	BMEI1339	Hypothetical protein	NC	3	[7]
BMEI1361	BMEI1361	Hypothetical protein	NC	33	[7]
BMEI1433	BMEI1433	Hypothetical protein	NC	56	[7]
BMEI1807	BMEI1807	Hypothetical protein	NC	53	[8]
BMEI1844	BMEI1844	Hypothetical protein	NC	17	[7]
BMEI1879	BMEI1879	Hypothetical protein	NC	4	[7]
BMEI0514	BMEI0514	Hypothetical protein	NC	1	[8]
BMEII0428	BMEII0428	D-erythrulose 4 phosphate dehydrogenase	NC	13	[8]
BMEI0455	BMEI0455	Glutathione S-transferase	O	198	[17]
BMEI0816	*clpA*	ATP-dependent Clp protease ATP-binding subunit	O	295	[8]
BMEI1049	*bcp*	Bacterioferritin comigratory protein	O	577	[24]
BMEI1069	*tig*	Trigger factor	O	660	[7]
BMEI1513	*dnaJ*	Molecular chaperone	O	113	[17]
BMEI1804	*glnD*	P II urydyl-transferase	O	315	[8]
BMEII0932	*nrdH*	Glutaredoxin	O	94	[8]
BMEI1327	*glnE*	Glutamine-synthetase adenylyltransferase	O/T	328	[8]
BMEI1766	*cysL*	Sulfite reductase (ferredoxin)	P	341	[17]
BMEII0056	*mgtB*	Magnesium ABC transporter ATPase	P	489	[17]
BMEI1167	BMEI1167	Hypothetical protein	Q	62	[7]
BMEI1143	*mbl*	Metal dependent hydrolase	R	500	[8]
BMEI1282	*gcvT*	Glycine cleavage system protein T	R	162	[8]
BMEI1443	BMEI1443	2-haloalkanoic acid dehalogenase	R	92	[17]
BMEI1487	*colV*	Colicin V production protein	R	174	[8]
BMEI1499	*pirA*	Pirin	R	326	[8]
BMEI1531	BMEI1531	Hypothetical protein	R	131	[7]
BMEI1867	*nifB/elp3*	Florfenicol resistance protein	R	604	[8]
BMEII0701	*rbsC*	Ribose ABC transporter permease	R	304	[17]
BMEII0039	*gltD*	Glutamate synthase subunit beta	R/E	0	[8]
BMEI0186	BMEI0186	Hypothetical protein	S	101	[8]
BMEI0330	*opgC*	Uncharacterized protein	S	68	[7]
BMEI0331	BMEI0331	Hypothetical protein	S	50	[7]
BMEI0490	BMEI0490	Hypothetical protein	S	70	[8]
BMEI0603	BMEI0603	Hypothetical protein	S	91	[8]
BMEI0732	BMEI0732	Hypothetical protein	S	183	[8]
BMEI1298	BMEI1298	Hypothetical protein	S	117	[8]
BMEI1809	BMEI1809	Hypothetical protein	S	66	[7]
BMEI1894	*grsT*	Gramicidin S biosynthesis protein	S	53	[8]
BMEII0128	BMEII0128	Hypothetical protein	S	54	[17]
BMEI1336	*phoQ*	Sensor protein	T	154	[7]
BMEI1448	BMEI1448	C-di-GMP phosphodiesterase A-related protein	T	28	[7], [19]
BMEI1453	*bpdA*	Diguanylate cyclase/phosphodiesterase	T	174	[19]
BMEII0011	*hydG*	Transcriptional regulator protein	T	55	[8]
BMEII0986	*nnrA*	CRP family transcriptional regulator	T	365	[15]
BMEI0066	*ompR*	Two component response regulator	T/K	81	[8], [23]
BMEI1296	BMEI1296	Guanosine-3′,5′-bis(diphosphate) 3′-pyrophosphohydrolase	T/K	635	[8]
BMEI1607	*prlR*	Glycerol metabolism activator	T/K	166	[18]
BMEII0025	*virB1*	Attachment mediating protein VIRB1-like protein	U	44	[8]
BMEII0026	*virB2*	Virb2	U	10	[8]
BMEII0027	*virB3*	Virb3	U	18	[8]
BMEII0028	*virB4*	ATPase VIRB4-like protein	U	129	[8], [20]
BMEII0029	*virB5*	Virb5	U	27	[8]
BMEII0030	*virB6*	Channel protein VIRB6-like protein	U	20	[8], [24]
BMEII0031	*virB7*	Virb7	U	2	[8]
BMEII0032	*virB8*	Virb8	U	67	[8], [24]
BMEII0033	*virB9*	Channel protein VIRB9-like protein	U	80	[8]
BMEII0034	*virB10*	Channel protein VIRB10-like protein	U	124	[8]
BMEI0926	*emrA*	Multidrug resistance protein A	V	1	[8]
BMEII0318	BMEII0318	6-aminohexanoate-dimer hydrolase, partial	V	62	[7]

Abbreviations: N.O.: number of orthologs in 789 non-redundant bacterial and archaeal genomes, COG: cluster of orthologous groups, A: RNA processing and modification, B: chromatin structure and dynamics, C: energy production and conversion, D: cell cycle control, cell division, chromosome partitioning, E: amino acid transport and metabolism, F: nucleotide transport and metabolism, G: carbohydrate transport and metabolism, H: coenzyme transport and metabolism, I: lipid transport and metabolism, J: translation, ribosomal structure and biogenesis, K: transcription, L: replication, recombination and repair, M: cell wall/membrane/envelope biogenesis, N: cell motility, NC: not in COG, O: posttranslational modifications, protein turnover, chaperones, P: inorganic ion transport and metabolism, Q: secondary metabolites biosynthesis, transport and catabolism, R: general function prediction only, S: function unknown, T: signal transduction mechanisms, U: intracellular trafficking, secretion and vesicular transport, V: defense mechanisms, W: extracellular structures.

### COG classification and comparison with the whole genome COG distribution

In order to have a better approximation of the functional annotations of the virulence-related genes described in previous studies, the genes were classified in terms of their COGs, and afterwards their frequencies were evaluated. From these analyses, we observed that 20 of the 24 COG categories were present in the virulence genes (Table 2). The most frequent COGs observed were genes for cell wall/membrane/envelope biogenesis (M) (10.62%), transcription (K) (10.44%), intracellular trafficking, secretion and vesicular transport (U) (7.81%) and carbohydrate transport and metabolism (G) (7.19%) while the least frequent COGs were secondary metabolites biosynthesis (Q) (0.62%), lipid transport and metabolism (I) (0.63%), defense mechanisms (V) (1.25%) and inorganic ion transport and metabolism (P) (1.88%) (Table 2). In the comparison of virulence genes with the whole-genome COG distribution, we found a significant overrepresentation of the COGs for intracellular trafficking, secretion and vesicular transport (U) (7.81% vs 1.5%), nucleotide transport and metabolism (F) (6.56% vs 2.2%), cell motility (N) (4.69% vs 0.92%), transcription (K) (10.44% vs 5.62%) and cell wall/membrane/envelope biogenesis (M) (10.62% vs 5.0%) and an underrepresentation of genes not categorized in a COG (NC) (Table 2). The difference was more significant for COGs U, N and F, which are composed mainly of genes grouped in the *virB* operon, flagellar genes and genes for enzymes related to nucleotide synthesis, respectively; COG M includes predominantly genes for enzymes related to lipopolysaccharide production, while COG K was mainly represented by RNA polymerase sigma factors and transcription factors of the families MerR, LysR, LuxR, AsnC and GntR (Table 1).

**Table 2:** Comparison of COG distribution in the virulence genes with the *B. melitensis* 16 M genome COG distribution.

COG	*B. melitensis* genome	Virulence genes	Comparison
	Frequency(%)	Frequency(%)	P value
A	1 (0.03)	0 (0)	1
B	1 (0.03)	0 (0)	1
C	172 (5.1)	10 (6.25)	0.51
D	27 (0.80)	0 (0)	0.63
E	326 (9.65)	9.5 (5.94)	0.15
F	74 (2.2)	10.5 (6.56)	0.001*
G	187 (5.53)	11.5 (7.19)	0.29
H	133 (3.93)	3.5 (2.19)	0.36
I	114 (3.37)	1 (0.63)	0.06
J	165 (4.88)	3.3 (2.06)	0.08
K	190 (5.62)	16.7 (10.44)	0.008*
L	107 (3.17)	5.5 (3.44)	0.68
M	169 (5.0)	17 (10.62)	0.002*
N	31 (0.92)	7.5 (4.69)	<0.001*
NC	573 (16.95)	11 (6.87)	<0.001*
O	121 (3.58)	8.5 (5.31)	0.18
P	157 (4.65)	3 (1.88)	0.09
Q	63 (1.86)	1 (0.62)	0.37
R	318 (9.41)	9 (5.62)	0.10
S	268 (7.94)	10 (6.25)	0.63
T	92 (2.72)	7 (4.38)	0.45
U	51 (1.50)	12.5 (7.81)	<0.001*
V	38 (1.13)	2 (1.25)	0.70
W	1 (0.03)	0 (0)	1
**Total**	**3379 (100)**	**160 (100)**	

P value obtained with chi-squared test and Fisher exact test,

*p-value ≤0.05. Abbreviations: COG: cluster of orthologous groups, A: RNA processing and modification, B: chromatin structure and dynamics, C: energy production and conversion, D: cell cycle control, cell division, chromosome partitioning, E: amino acid transport and metabolism, F: nucleotide transport and metabolism, G: carbohydrate transport and metabolism, H: coenzyme transport and metabolism, I: lipid transport and metabolism, J: translation, ribosomal structure and biogenesis, K: transcription, L: replication, recombination and repair, M: cell wall/membrane/envelope biogenesis, N: cell motility, NC: not in COG, O: posttranslational modifications, protein turnover, chaperones, P: inorganic ion transport and metabolism, Q: secondary metabolites biosynthesis, transport and catabolism, R: general function prediction only, S: function unknown, T: signal transduction mechanisms, U: intracellular trafficking, secretion and vesicular transport, V: defense mechanisms, W: extracellular structures.

### Taxonomical distribution and comparison with random genes

We observed a significantly higher number of orthologs among the virulence genes in comparison with random genes (p = 0.034). The virulence genes presented a median (range) of 174 (0–727) orthologs and 119 (0–788) for random genes. These data were obtained by adding all the orthologs of each gene included and then obtaining and comparing (with the Mann-Whitney U test) the median and range per group of genes (random and virulence). In Table 3 we show the taxonomical distribution of the orthologs present among the virulence and random genes. These analyses were conducted by comparing the frequencies and percentages of the number of orthologs in each organism division and not the numerical count (i.e., medians and ranges). We used this method after considering that the presence or absence of an ortholog is a dichotomous qualitative variable and the best way to analyze its behavior is by using qualitative probes, which better describe their distribution in the different bacterial and archaeal divisions, thus avoiding a possible bias by the total increase or decrease of orthologs in a particular set of genes. By using the frequencies and percentages, we were able to identify an increase or a decrease in the proportion with respect to the total number of orthologs in a particular bacterial or archaeal division with higher accuracy than when using quantitative probes.

**Table 3:** Comparison of the taxonomic distribution of virulence genes and randomly selected genes of *B. melitensis* 16 M in bacterial and archaeal groups.

Cellular Domain	Taxonomical classification	Random genes (N = 250)	Virulence genes (N = 160)	Comparison
**Archaea**	Phylum (N)	N, % (A.P.)	N, % (A.P.)	P value
	*Crenarchaeota* (25)	595, 1.15 (1.72)	447, 1.18 (1.84)	0.71
	*Euryarchaeota* (61)	1649, 3.19 (1.84)	1366, 3.60 (2.22)	<0.001*
	*Korarchaeota* (1)	23, 0.04 (2.20)	11, 0.03 (1.53)	0.24
	*Nanoarchaeota* (1)	5, 0.01 (1.43)	2, 0.01 (0.85)	0.70
	*Thaumarchaeota* (1)	24, 0.05 (2.04)	21, 0.05 (2.60)	0.56
**Bacteria**	*Acidobacteria* (3)	241, 0.47 (2.68)	173, 0.46 (2.77)	0.81
	*Actinobacteria* (90)	6075, 11.76 (3.00)	4527, 11.93 (3.36)	0.42
	*Aquificae* (5)	263, 0.51(4.72)	194, 0.51 (5.07)	0.96
	*Bacteroidetes* (30)	1687, 3.27 (3.35)	1122, 2.96 (3.05)	<0.01*
	*Chlamydiae* (8)	253, 0.49 (4.18)	129, 0.34 (3.10)	<0.001*
	*Chlorobi* (11)	665, 1.29 (4.08)	461, 1.22 (4.10)	0.34
	*Chloroflexi* (12)	627, 1.21 (3.59)	425, 1.12 (3.39)	0.20
	*Cyanobacteria* (31)	1787, 3.46 (2.86)	1280, 3.38 (2.99)	0.49
	*Deferribacteres* (3)	200, 0.39 (4.23)	155, 0.41 (4.76)	0.61
	*Deinococcus-Thermus* (3)	202, 0.39 (3.71)	137, 0.36 (3.66)	0.47
	*Dictyoglomi* (2)	104, 0.20 (4.35)	77, 0.20 (4.70)	0.96
	*Elusimicrobia* (1)	44, 0.09 (4.39)	33, 0.09 (4.80)	0.93
	*Fibrobacteres* (1)	62, 0.12 (3.07)	36, 0.09 (2.60)	0.26
	*Firmicutes* (120)	6956, 13.47 (3.19)	4902, 12.93 (3.25)	0.02*
	*Fusobacteria* (5)	253, 0.49 (3.27)	182, 0.48 (3.42)	0.83
	*Gemmatimonadetes* (1)	66, 0.13 (2.56)	52, 0.14 (2.93)	0.70
	*Nitrospirae* (1)	57, 0.11 (4.39)	43, 0.11 (4.82)	0.89
	*Planctomycetes* (3)	192, 0.37 (1.90)	135, 0.36 (1.95)	0.70
	*Proteobacteria* (316)	27661, 53.56 (4.38)	20794, 54.83 (4.61)	<0.001*
	*Spirochaetes* (15)	680, 1.32 (3.59)	455, 1.2 (3.26)	0.12
	*Synergistetes* (2)	99, 0.19 (4.20)	78, 0.21 (4.84)	0.64
	*Tenericutes* (25)	507, 0.98 (4.38)	269, 0.71 (3.38)	<0.0001*
	*Thermobaculum* (1)	64, 0.12 (3.45)	47, 0.12 (3.69)	0.99
	*Thermotogae* (7)	357, 0.69 (4.06)	219, 0.58 (3.64)	0.04*
	*Verrucomicrobia* (4)	247, 0.48 (3.19)	150, 0.39 (2.82)	0.07
	**Total (789)**	**51645, 100 (100)**	**37922, 100 (100)**	

N =  Number, A.P.: adjusted percentage, this is the corresponding percentage of the number of orthologs per each 100 genes of each organisms and divided by the number of organisms in each group, P value obtained with chi-squared test and Fisher exact test, *p value ≤0.05. In order to understand the differences in the percentages between the groups, the percentage and not the adjusted percentage must be taken into account, the adjusted percentage should only be considered in descriptive results, i.e., to know the distribution of orthologs in the organisms groups in each set of genes.

In order to know the distribution of the orthologs in the 2 sets of genes in the different bacterial and archaeal genomes, we adjusted the percentages of the observed orthologs by dividing the total number of orthologs for each organism by the number of genes in its genome, and the sum of these values in one particular division was divided by the number of organisms analyzed in that division. Finally we adjusted the total to 100% and obtained the corresponding percentage by group of organisms. After adjustments by genome size and number of organisms analyzed per group, we observed that the highest proportions of orthologs in random genes were found in *Aquificae*, *Nitrospirae*, *Elusimicrobia* and *Proteobacteria*, and the lowest frequencies were in *Nanoarchaeota*, *Crenarchaeota* and *Euryarchaeota*. Virulence genes presented the highest frequencies in *Aquificae, Synergistetes, Nitrospirae* and *Deferribacteres* and the lowest frequencies in the archaeal groups *Nanoarchaeota, Korarchaeota* and *Crenachaeota* (Table 3).

In the comparison of virulence genes with the randomly selected genes, we observed a slight but significant increase of orthologs in *Euryarchaeota* and *Proteobacteria*, with a significant decrease in *Tenericutes, Chlamydiae, Bacteroidetes, Firmicutes* and *Thermotogae*. The differences within the *Proteobacteria*, and the *Alphaproteobacteria* groups are described in Table 4. Within *Proteobacteria* division, in virulence genes, we found a slight but significant decrease of orthologs in *Gammaproteobacteria*, and within *Alphaproteobacteria* we found a significant decrease in the order of *Rickettsiales* when compared with randomly selected genes. It is important to mention that in order to observe the differences in the comparisons of the orthologs present, the absolute percentage and not the adjusted percentage must be considered, because for comparison purposes the number of genes per genome or the number of genomes by each particular division did not affect the differences observed, as the same organisms were used to obtain the orthologs in the 2 sets of genes. This is why these percentages are more accurate and better reflect the differences between both groups. The adjusted percentages must be taken into account only to appreciate the orthologs distribution in each set of genes, as previously mentioned.

**Table 4:** Comparison of the taxonomic distribution of virulence genes and randomly selected genes of *B. melitensis* 16 M within *Proteobacteria* group.

Bacteria group	Taxonomical classification	Random genes (N = 250)	Virulence genes (N = 160)	Comparison
***Proteobacteria***	**Phylum (N)**	**N, % (A.P.)**	**N, % (A.P.)**	**P value**
	*Alphaproteobacteria* (92)	9691, 35.03 (26.08)	7407, 35.62 (26.24)	0.18
	*Betaproteobacteria* (54)	5057, 18.28 (18.16)	3851, 18.52 (18.66)	0.50
	*Deltaproteobacteria* (29)	2148, 7.77 (14.87)	1704, 8.19 (15.84)	0.08
	*Epsilonproteobacteria* (21)	1086, 3.93 (19.57)	846, 4.07 (20.31)	0.43
	*Gammaproteobacteria* (120)	9679, 34.99 (21.32)	6986, 33.60 (18.95)	0.001*
	**Total (316)**	27661, 100 (100)	20794, 100 (100)	
***Alphaproteobacteria***	*Caulobacterales* (5)	589, 6.08 (9.54)	464, 6.26 (10.14)	0.62
	*Magnetococcales* (1)	76, 0.78 (6.86)	59, 0.80 (7.15)	0.93
	*Parvularculares* (1)	103, 1.06 (12.51)	76, 1.03 (12.42)	0.81
	*Rhizobiales* (37)	4841, 49.95 (10.44)	3732, 50.38 (10.92)	0.58
	*Rhodobacterales* (11)	1375, 14.19 (10.33)	1062, 14.34 (10.77)	0.78
	*Rhodospirillales* (9)	1000, 10.32 (10.97)	792, 10.69 (11.81)	0.43
	*Rickettsiales* (20)	941, 9.71 (14.68)	634, 8.56 (13.36)	<0.01*
	*Sphingomonadales* (6)	597, 6.16 (9.98)	477, 6.44 (10.73)	0.46
	Unclassified (2)	169, 1.75 (14.69)	111, 1.50 (12.70)	0.21
	**Total (92)**	9691, 100 (100)	7407, 100 (100)	

N =  Number, A.P.: adjusted percentage, this is the corresponding percentage of the number of orthologs per each 100 genes of each organisms and divided by the number of organisms in each group, P value obtained with chi-squared test and Fisher exact test,

*p value ≤0.05. In order to understand the differences in the percentages between the groups, the percentage and not the adjusted percentage must be taken into account, the adjusted percentage should only be considered in descriptive results, i.e., to know the distribution of orthologs in the organisms groups in each set of genes.

The virulence genes with the lowest number of orthologs were BMEII0039 (*gltD*) and BMEI0972 (*gor*), with no orthologs, followed by BMEI0998 (*wboA*), BMEI0926 (*emrA*) and the three hypothetical proteins BMEI0540, BMEI0514 and BMEI0193, with only one ortholog (in *B. suis*). The gene BMEII0031 (*virB7*) only presented 2 orthologs (in *B. suis* and *Methylobacterium radiotolerans* JCM1831) (Table 1). The genes with the highest number of orthologs were BMEI1301 (*dapA*), BMEI0296 (*purE*), BMEI1519 (*purD*), BMEI1127 (*purL*) and BMEI0781 (*rpoA*), with around 700 orthologs (Table 1). Additionally, orthologs of the *virB* operon were found in different organisms; nevertheless, in addition to *B. suis* (the only member of the family *Brucelaceae* included), *Methylobacterium radiotolerans* JCM1831 was the only analyzed bacterium that presented an ortholog for the 11 genes of this operon, followed by *Burkholderia vietnamiensis* G4, *B. xenovorans* LB400 and *Leptothrix cholodnii* SP6, with 10 *virB* genes.

## Discussion

In this report we comprehensively evaluated all the attenuated mutants in *B. melitensis* described in the literature. Additionally, we performed a COG and taxonomical comparison in order to gain insights into the function and distribution of these genes.

From these analyses we observed virulence genes in all but 4 COGs, which indicate that virulence genes are present in almost all functional categories. The absence of virulence genes in COGs A, B, D and W is probably due to the extremely low frequencies of these COGs in the *B. melitensis* genome (<1%). In the comparison of the most highly represented COGs among the virulence genes, we found good agreement with the largest study performed in random mutants of *B. melitensis* [8], in which COGs related to intracellular trafficking and vesicular transport (U), nucleotide transport and metabolism (F), transcription (K), cell wall/membrane/envelope biogenesis (M) and energy production and conversion (C) were overrepresented in attenuated mutants. In this study, where the information was complemented with information on the rest of the mutants reported so far for *B. melitensis*, we also found that COG N (composed of flagellar genes) was overrepresented, while COG C was no more significantly affected (Table 2). These results showed that genes related to intracellular trafficking, mainly composed of *virB* and flagellar genes and also to genes related to nucleotide synthesis, lipopolysaccharide production and transcription factors of the families MerR, LysR, LuxR, AsnC and GntR, are essential for *B. melitensis* virulence. These observations have been mainly confirmed for *virB* genes, for which many independent studies have shown that *virB* is required for infection persistence [8], [11], [20], [24]. As mutants lacking this operon show normal dissemination, it has been proposed that this genes products are not required in early infection establishment but instead in the late stages of infection [20]. Some mutants classified in the COGs related to transcription (K), such as RNA polymerase sigma factors and VjbR and ArsR6 transcription factors, are associated with virulence based on their importance in *virB* transcription (Table 1). The underrepresentation of genes not categorized in a COG suggests that uncharacterized genes are less relevant in *B. melitensis* virulence.

The increased number of orthologs for virulence genes compared with randomly selected genes suggests an increased conservation of these genes in different bacterial and archaeal groups, which is probably related to a higher importance of their functions in the analyzed organisms. It is important to mention that some of these genes affect the intracellular replication of *B. melitensis* by disruption of general metabolism (i.e., nucleotide metabolism), which is also essential in other nonpathogenic organisms, and so these genes could be contributing to the higher number of orthologs found compared to results with the randomly selected genes.

The adjusted taxonomical distribution of orthologs among virulence and random genes was unexpectedly found with a high frequency in bacterial groups different from *Proteobacteria*, including *Aquificae*, *Nitrospirae, Deferribacteres* and *Elusimicrobia* (Table 3). This could be explained by events of massive horizontal transfer between the *Brucellaceae* family and these groups, as proposed in deviations from the genome-wide molecular clock [18], although it is also important to consider that these groups are composed of a small number of organisms, which diminishes the representativeness and could affect their true distribution. The significantly high number of orthologs in *Euryarchaeota*, which was even more pronounced in *Proteobacteria*, together with the low proportion of orthologs in other bacterial groups, including *Tenericutes, Chlamydiae, Bacteroidetes, Firmicutes* and *Thermotogae*, indicates that virulence genes are more conserved in *Euryarchaeota* (*Archaea*) and *Proteobacteria* than expected by chance, with an underrepresentation of many bacterial groups, including intracellular microorganisms (Table 3). These observations seem contradictory, considering that it is thought that virulence genes are more likely to be associated with intracellular adaptations; nevertheless, it has been shown that some virulence genes in *Brucella* spp. are also needed for plant symbiosis or even plant virulence in members of the *Rhizobiaceae* family, a phylogenetically related family with a different lifestyle (plant symbionts or plant pathogens) [27], [28], [29]. This suggests an evolution and adaptation of these genes ancestors to different environments, such as animal or plant intracellular life. To date, the main virulence genes associated with intracellular survival in *B. melitensis* are composed of the *virB* operon, which is more closely related to *Proteobacteria* than to other bacterial groups. Other virulence-related genes reported so far exhibit a wide diversity of functions, including transcription, membrane structure and cell motility, which are more likely to be associated with phylogenetically related organisms than with distantly related ones when compared with randomly selected genes. The significant diminishment in the number of orthologs in *Gammaproteobacteria* and *Rickettisales* (Table 4) suggests a diminished conservation of these genes in these particular groups within the *Proteobacteria* and the *Alphapoteobacteria* groups, respectively, which could also be explained by gene loss in these specific divisions. It is important to mention that the proportion of orthologs in virulence genes in the *Alphaproteobacteria* was not increased within the *Proteobacteria* group; likewise, there was not an increased proportion of orthologs in the *Rhizobiales* (the order to which *B. melitensis* belongs) within the *Alphaproteobacteria* subgroup. These results could indicate that although there is not an increased proportion of orthologs in these subgroups, the increased number of orthologs in the *Proteobacteria* suggests that a higher number of orthologs is distributed in all the subgroups of *Proteobacteria* in a similar proportion, including the *Alphaproteobacteria* and hence the *Rhizobiales*, with the exception of *Gammaproteobacteria* and *Rickettsiales*, which presented a diminished presence (as previously mentioned). The lack of a particular increase of orthologs in *Alphaproteobacteria* and *Rhizobiales* could be due to a nonpreferential conservation of these genes in these subgroups and/or that the slight increase observed (Table 4) does not reach statistical significance due to the small sample size, considering that these comparisons were performed with a smaller sample size than the comparisons for the main groups of organisms.

With respect to the number of orthologs present in specific virulence genes, we found that genes related to specialized functions, such as BMEII0039 (*gltD*), BMEI0972 (*gor*), BMEI0998 (*wboA*) and BMEI0926 (*emrA*), as well as 3 hypothetical proteins with unknown function (BMEI0540, BMEI0514 and BMEI0193) exhibited the lowest number of orthologs, suggesting a set of unique genes involved in the *B. melitensis* virulence network that could be candidates for specific drug targets. While the genes with known functions that are important but more general, such as nucleotide metabolism [BMEI0296 (*purE*), BMEI1519 (*purD*) and BMEI1127 (*purL*)] and the RNA polymerase BMEI0781 (*rpoA*), presented the highest number of orthologs. It is noteworthy that the gene with the highest number of orthologs was BMEI1301 (*dapA*), a gene involved in lysine biosynthesis (Table 1).

Considering the few organisms with orthologs for the complete set of genes included in the *virB* operon (besides *B. suis*), we propose a possible mechanism of horizontal transfer or a similar evolution to other *Rhizobiales* (in the case of *Methylobacterium radiotolerans*) or even *Betaproteobacteria* (in the case of *Burkholderia vietnamensis, B. xenovorans* and *Leptothrix cholodnii*), as these bacteria are not the closest organisms to *B. melitensis* that were analyzed. A mechanism of gene loss in closer organisms is also possible.

In conclusion, 160 virulence-related genes in *B. melitensis* were retrieved from the literature. They exhibited a wide range of functions with an overrepresentation of specific COGs, such as intracellular trafficking and vesicular transport, transcription, cell wall and membrane biogenesis, nucleotide transport and metabolism and cell motility.

The taxonomical distribution analysis showed low but significant differences between virulence and randomly selected genes in *B. melitensis*. These differences could be related to the conservation of these genes in different bacterial groups and are probably related to their functions (considering that a higher conservation is closely related to a more required function). In this line, the high number of orthologs in *Proteobacteria* and to a lesser extent in *Euryarchaeota* (from the *Archaea* domain) with a significant decrease in other groups when compared with randomly selected genes is remarkable and indicates a higher conservation of these genes within the *Proteobacteria* group, which in the case of *B. melitensis* required adaptation to animal intracellular pathogenicity. These differences would also indicate a reduced likelihood of horizontal transfer with distant related organisms with the exception of *Euryarchaeota*; nevertheless, further experimental and integrative analyses will redefine the main functions and taxonomical distribution of virulence genes in *B. melitensis*. Finally, our integrative approach suggests potential specific drug targets, such as the *gltD, gor* and *wboA* genes, that when combined with drugs that affect virulence genes of different functions could be used in a synergistic fashion to treat *B. melitensis* infection.

## Supporting Information

Table S1 BMEI locus and functional description of randomly selected genes.
(DOCX)

## References

[1] Uzureau S, Godefroid M, Deschamps C, Lemaire J, De Bolle X, et al (2007) Mutations of the quorum sensing-dependent regulator VjbR lead to drastic surface modifications in Brucella melitensis. J Bacteriol 189: 6035–6047. 17557825 10.1128/JB.00265-07 PMC1952030

[2] Uzureau S, Lemaire J, Delaive E, Gaigneaux A, Raes M, et al (2010) Global analysis of quorum sensing targets in the intracellular pathogen Brucella melitensis 16 M. J Proteome Res 9: 3200–3217. 20387905 10.1021/pr100068p PMC2880877

[3] Dokuzoğuz B, Ergönül O, Baykam N, Esener H, Kiliç S, et al (2005) Characteristics of B. melitensis versus B. abortus bacteraemias. J Infect 50: 41–45. 15603839 10.1016/j.jinf.2004.02.005

[4] Troy SB, Rickman LS, Davis CE (2005) Brucellosis in San Diego: epidemiology and species-related differences in acute clinical presentations. Medicine (Baltimore) 84: 174–187. 15879907 10.1097/01.md.0000165659.20988.25

[5] Baldi PC, Giambartolomei GH (2013) Immunopathology of Brucella infection. Recent Pat Antiinfect Drug Discov 8: 18–26. 22812614 10.2174/1574891x11308010005

[6] He Y (2012) Analyses of Brucella pathogenesis, host immunity, and vaccine targets using systems biology and bioinformatics. Front Cell Infect Microbiol 2: 2. 22919594 10.3389/fcimb.2012.00002 PMC3417401

[7] Lestrate P, Dricot A, Delrue RM, Lambert C, Martinelli V, et al (2003) Attenuated signature-tagged mutagenesis mutants of Brucella melitensis identified during the acute phase of infection in mice. Infect Immun 71: 7053–7060. 14638795 10.1128/IAI.71.12.7053-7060.2003 PMC308902

[8] Wu Q, Pei J, Turse C, Ficht TA (2006) Mariner mutagenesis of Brucella melitensis reveals genes with previously uncharacterized roles in virulence and survival. BMC Microbiol 6: 102. 17176467 10.1186/1471-2180-6-102 PMC1766931

[9] Fretin D, Fauconnier A, Köhler S, Halling S, Léonard S, et al (2005) The sheathed flagellum of Brucella melitensis is involved in persistence in a murine model of infection. Cell Microbiol 7: 687–698. 15839898 10.1111/j.1462-5822.2005.00502.x

[10] Edmonds MD, Cloeckaert A, Elzer PH (2002) Brucella species lacking the major outer membrane protein Omp25 are attenuated in mice and protect against Brucella melitensis and Brucella ovis. Vet Microbiol 88: 205–221. 12151196 10.1016/s0378-1135(02)00110-4

[11] Nijskens C, Copin R, De Bolle X, Letesson JJ (2008) Intracellular rescuing of a B. melitensis 16 M virB mutant by co-infection with a wild type strain. Microb Pathog 45: 134–141. 18547782 10.1016/j.micpath.2008.04.005

[12] Rajashekara G, Covert J, Petersen E, Eskra L, Splitter G (2008) Genomic island 2 of Brucella melitensis is a major virulence determinant: functional analyses of genomic islands. J Bacteriol 190: 6243–6252. 18641138 10.1128/JB.00520-08 PMC2546784

[13] Crawford RM, Van De Verg L, Yuan L, Hadfield TL, Warren RL, et al (1996) Deletion of purE attenuates Brucella melitensis infection in mice. Infect Immun 64: 2188–2192. 8675325 10.1128/iai.64.6.2188-2192.1996 PMC174054

[14] Delory M, Hallez R, Letesson JJ, De Bolle X (2006) An RpoH-like heat shock sigma factor is involved in stress response and virulence in Brucella melitensis 16 M. J Bacteriol 188: 7707–7010. 16936018 10.1128/JB.00644-06 PMC1636281

[15] Haine V, Dozot M, Dornand J, Letesson JJ, De Bolle X (2006) NnrA is required for full virulence and regulates several Brucella melitensis denitrification genes. J Bacteriol 188: 1615–1619. 16452445 10.1128/JB.188.4.1615-1619.2006 PMC1367225

[16] Haine V, Sinon A, Van Steen F, Rousseau S, Dozot M, et al (2005) Systematic targeted mutagenesis of Brucella melitensis 16 M reveals a major role for GntR regulators in the control of virulence. Infect Immun 9: 5578–5586. 10.1128/IAI.73.9.5578-5586.2005 PMC1231144 16113274

[17] Lestrate P, Delrue RM, Danese I, Didembourg C, Taminiau B, et al (2000) Identification and characterization of in vivo attenuated mutants of Brucella melitensis. Mol Microbiol 38: 543–551. 11069678 10.1046/j.1365-2958.2000.02150.x

[18] Mirabella A, Yañez RM, Villanueva RM, Delrue RM, Uzureau S, et al (2012) The two-component system PrlS/PrlR of Brucella melitensis is required for persistence in mice and appears to respond to ionic strength. Microbiology 158: 2642–2651. 22859617 10.1099/mic.0.060863-0

[19] Petersen E, Chaudhuri P, Gourley C, Harms J, Splitter G (2011) Brucella melitensis cyclic di-GMP phosphodiesterase BpdA controls expression of flagellar genes. J Bacteriol 193: 5683–5691. 21856843 10.1128/JB.00428-11 PMC3187190

[20] Rajashekara G, Glover DA, Banai M, O'Callaghan D, Splitter GA (2006) Attenuated bioluminescent Brucella melitensis mutants GR019 (virB4), GR024 (galE), and GR026 (BMEI1090-BMEI1091) confer protection in mice. Infect Immun 74: 2925–2936. 16622231 10.1128/IAI.74.5.2925-2936.2006 PMC1459743

[21] Rambow-Larsen AA, Rajashekara G, Petersen E, Splitter G (2008) Putative quorum-sensing regulator BlxR of Brucella melitensis regulates virulence factors including the type IV secretion system and flagella. J Bacteriol 190: 3274–3282. 18310341 10.1128/JB.01915-07 PMC2347389

[22] Wang Z, Niu J, Wang S, Lv Y, Wu Q (2013) In vivo differences in the virulence, pathogenicity, and induced protective immunity of wboA mutants from genetically different parent Brucella spp. Clin Vaccine Immunol 20: 174–180. 23239800 10.1128/CVI.00573-12 PMC3571281

[23] Zhang X, Ren J, Li N, Liu W, Wu Q (2009) Disruption of the BMEI0066 gene attenuates the virulence of Brucella melitensis and decreases its stress tolerance. Int J Biol Sci 5: 570–577. 19742243 10.7150/ijbs.5.570 PMC2737717

[24] Zygmunt MS, Hagius SD, Walker JV, Elzer PH (2006) Identification of Brucella melitensis 16 M genes required for bacterial survival in the caprine host. Microbes Infect 8: 2849–2854. 17090391 10.1016/j.micinf.2006.09.002

[25] Janga SC, Moreno-Hagelsieb G (2004) Conservation of adjacency as evidence of paralogous operons. Nucleic Acids Res 32: 5392–5397. 15477389 10.1093/nar/gkh882 PMC524292

[26] Novichkov PS, Omelchenko MV, Gelfand MS, Mironov AA, Wolf YI, et al (2004) Genome-wide molecular clock and horizontal gene transfer in bacterial evolution. J Bacteriol 186: 6575–6585. 15375139 10.1128/JB.186.19.6575-6585.2004 PMC516599

[27] Guzman-Verri C, Manterola L, Sola-Landa A, Parra A, Cloeckaert A, et al (2002) The two-component system BvrR/BvrS essential for Brucella abortus virulence regulates the expression of outer membrane proteins with counterparts in members of the Rhizobiaceae. Proc Natl Acad Sci USA 99: 12375–12380. 12218183 10.1073/pnas.192439399 PMC129452

[28] Iñón de Iannino N, Briones G, Tolmasky M, Ugalde RA (1998) Molecular cloning and characterization of cgs, the Brucella abortus cyclic beta(1–2) glucan synthetase gene: genetic complementation of Rhizobium meliloti ndvB and Agrobacterium tumefaciens chvB mutants. J Bacteriol 180: 4392–4400. 9721274 10.1128/jb.180.17.4392-4400.1998 PMC107446

[29] LeVier K, Phillips RW, Grippe VK, Roop RM 2nd, Walker GC (2000) Similar requirements of a plant symbiont and a mammalian pathogen for prolonged intracellular survival. Science 287: 2492–2493. 10741969 10.1126/science.287.5462.2492

