# Supplementary material for: Main Functions and Taxonomic Distribution of Virulence Genes in Brucella melitensis 16 M
Source: PLoS One. 2014 Jun 25;9(6):e100349. doi: 10.1371/journal.pone.0100349 (PMC4070974; doi:10.1371/journal.pone.0100349)
Supplement: Table S1 — BMEI locus and functional description of randomly selected genes. (DOCX) [file pone.0100349.s001.docx]

**Supplementary Table.** BMEI locus and functional description of randomly selected genes.

| **BMEI locus** | **Functional description** |
| --- | --- |
| BMEI1945 | Enoyl-coa hydratase |
| BMEII0580 | Probable blue-copper protein yack precursor |
| BMEI0137 | Malate dehydrogenase |
| BMEI0201 | Lsu ribosomal protein l21p |
| BMEI0847 | Protein-export membrane protein |
| BMEII1123 | Iron(III)-transport atp-binding protein sfuc |
| BMEII1118 | Multidrug resistance protein a |
| BMEII1117 | Transcriptional regulator, tetr family |
| BMEII1078 | Hypothetical protein |
| BMEII1061 | (S)-2-hydroxy-acid oxidase subunit glcf |
| BMEII1059 | Protein ybis precursor |
| BMEII1056 | Histidyl-trna synthetase |
| BMEII1036 | Zinc protease |
| BMEII1017 | Mercuric resistance operon regulatory protein |
| BMEII0982 | Galactoside transport atp-binding protein mgla |
| BMEII0971 | Copper transport atp-binding protein nosf |
| BMEII0953 | Respiratory nitrate reductase 2 gamma chain |
| BMEII0948 | Nitrite extrusion protein |
| BMEII0944 | Maltose transport system permease protein malf |
| BMEII0939 | Thua |
| BMEII0912 | Hypothetical protein |
| BMEII0904 | Rtn protein |
| BMEII0897 | Chloride channel protein |
| BMEII0882 | Nitrogen fixation protein vnfa |
| BMEII0875 | Leucine-specific binding protein precursor |
| BMEII0868 | Leucine-specific binding protein precursor |
| BMEII0858 | Transcriptional regulator, gntr family |
| BMEII0854 | Transcription regulator, crp family |
| BMEII0850 | Gdp-fucose synthetase |
| BMEII0837 | Glycosyl transferase |
| BMEII0835 | Glycosyl transferase |
| BMEII0820 | Als operon regulatory protein |
| BMEII0788 | Hypothetical protein |
| BMEII0780 | Hypothetical protein |
| BMEII0772 | Hypothetical cytosolic protein |
| BMEII0755 | Sugar-binding protein |
| BMEII0754 | Sugar-binding protein |
| BMEII0725 | Hypothetical protein |
| BMEII0713 | Transposase |
| BMEII0708 | Ebsc protein |
| BMEII0704 | Bacterioferritin |
| BMEII0677 | Transporter, dme family |
| BMEII0672 | Dna integration/recombination/invertion protein |
| BMEII0661 | 50s ribosomal protein l33 |
| BMEII0651 | Hypothetical protein |
| BMEII0640 | 4-hydroxybenzoate 3-monooxygenase |
| BMEII0620 | Sulfur deprivation response regulator |
| BMEII0617 | Xanthine/uracil permease |
| BMEII0612 | Branched-chain amino acid aminotransferase |
| BMEII0589 | Riboflavin synthase subunit beta |
| BMEII0574 | Myo-inositol 2-dehydrogenase |
| BMEII0536 | Iron(iii) dicitrate transport system permease protein fecd |
| BMEII0533 | Fusaric acid resistance protein fuse |
| BMEII0528 | Glutamate-cysteine ligase |
| BMEII0524 | Hypothetical protein |
| BMEII0522 | Hypothetical protein |
| BMEII0477 | Uronate isomerase |
| BMEII0476 | Uronate isomerase |
| BMEII0463 | Putative icc-like phosphoesterase |
| BMEII0462 | Atp-dependent helicase |
| BMEII0448 | Zinc metallopeptidase |
| BMEII0434 | Hypothetical protein |
| BMEII0402 | Atp-dependent protease la 2 |
| BMEII0359 | Multiple sugar-binding periplasmic receptor chve precursor |
| BMEII0299 | Transcriptional regulator, iclr family |
| BMEII0252 | Methylglyoxal synthase |
| BMEII0205 | Dipeptide transport atp-binding protein dppf |
| BMEII0201 | Oligopeptide transport system permease protein oppc |
| BMEII0200 | Oligopeptide transport atp-binding protein oppd |
| BMEII0190 | Hypothetical protein |
| BMEII0185 | D-lactate dehydrogenase |
| BMEII0178 | High-affinity zinc uptake system protein znua |
| BMEII0177 | High-affinity zinc uptake system atp-binding protein znuc |
| BMEII0164 | Flagellar basal body rod modification protein |
| BMEII0149 | Extracellular serine protease |
| BMEII0139 | Phosphotyrosyl phosphatase activator (ptpa) |
| BMEII0126 | Amino acid permease |
| BMEII0015 | Homospermidine synthase |
| BMEI2057 | Dephospho-coa kinase |
| BMEI2052 | Membrane-bound lytic murein transglycosylase a |
| BMEI2050 | Transcriptional regulator |
| BMEI2028 | Hypothetical protein |
| BMEI2013 | Hypothetical protein |
| BMEI2012 | Benzoate membrane transport protein |
| BMEI2004 | Phenylalanyl-trna synthetase beta subunit |
| BMEI1991 | Hypothetical protein |
| BMEI1990 | Hypothetical membrane spanning protein |
| BMEI1983 | Spore-cortex-lytic enzyme prepeptide precursor |
| BMEI1977 | 1-acyl-sn-glycerol-3-phosphate acyltransferase |
| BMEI1967 | Transcription elongation factor nusa |
| BMEI1958 | Enoyl-(acyl carrier protein) reductase |
| BMEI1956 | 3-hydroxydecanoyl-acp dehydratase |
| BMEI1955 | Ferric uptake regulation protein |
| BMEI1944 | 30s ribosomal protein s20 |
| BMEI1927 | Enoyl-coa hydratase |
| BMEI1909 | Hypothetical protein |
| BMEI1903 | Cytochrome c-552 |
| BMEI1900 | Cytochrome o ubiquinol oxidase subunit i |
| BMEI1884 | Dienelactone hydrolase and related enzyme |
| BMEI1863 | Low molecular weight phosphotyrosine protein phosphatase |
| BMEI1849 | Thiol:disulfide interchange protein cycy precursor |
| BMEI1825 | Dna polymerase i |
| BMEI1816 | Sensory transduction protein kinase |
| BMEI1815 | Transposase |
| BMEI1814 | Transposase |
| BMEI1794 | Integration host factor beta subunit |
| BMEI1777 | Grpe protein |
| BMEI1772 | Putative deoxyribonucleotide triphosphate pyrophosphatase |
| BMEI1769 | Hypothetical protein |
| BMEI1767 | Hypothetical protein |
| BMEI1765 | Phosphoadenosine phosphosulfate reductase |
| BMEI1741 | Hypothetical protein |
| BMEI1715 | Maltose transport system permease protein malf |
| BMEI1698 | Hypothetical protein |
| BMEI1684 | Hypothetical protein |
| BMEI1677 | Hypothetical protein |
| BMEI1669 | Hypothetical protein |
| BMEI1661 | Recombinase |
| BMEI1656 | Hypothetical protein |
| BMEI1641 | Transcriptional regulator, tetr family |
| BMEI1637 | Coxg protein |
| BMEI1599 | Hypothetical protein |
| BMEI1565 | Cytochrome c oxidase, monoheme subunit, membrane-bound |
| BMEI1534 | Methyltransferase |
| BMEI1503 | Exodeoxyribonuclease vii small subunit |
| BMEI1501 | Transglycosylase associated protein |
| BMEI1484 | Cyclopropane-fatty-acyl-phospholipid synthase |
| BMEI1476 | Hypothetical protein |
| BMEI1473 | 3-oxoacyl-(acyl carrier protein) synthase |
| BMEI1451 | Processing peptidase |
| BMEI1447 | Hypothetical protein |
| BMEI1446 | Phosphoglycolate phosphatase |
| BMEI1432 | Hydroxyacylglutathione hydrolase |
| BMEI1431 | Bioy protein |
| BMEI1410 | Transposase |
| BMEI1407 | Transposase |
| BMEI1405 | Transposase |
| BMEI1399 | Transposase |
| BMEI1398 | Transposase |
| BMEI1394 | Mannose-6-phosphate isomerase |
| BMEI1390 | D-ribose-binding periplasmic protein precursor |
| BMEI1362 | Hypothetical protein |
| BMEI1350 | Phage dna packaging protein |
| BMEI1349 | Phage portal protein |
| BMEI1335 | 17 kd surface antigen precursor |
| BMEI1334 | Cytochrome c-type biogenesis protein cych |
| BMEI1310 | Hypothetical membrane spanning protein |
| BMEI1303 | Hypothetical cytosolic protein |
| BMEI1287 | Ribonuclease iii |
| BMEI1237 | Udp-glucose 4-epimerase |
| BMEI1226 | Transcriptional regulator |
| BMEI1213 | Cystathionine beta-lyase |
| BMEI1202 | Aspartyl-trna synthetase |
| BMEI1192 | Serine hydroxymethyltransferase |
| BMEI1183 | Hypothetical protein |
| BMEI1146 | Nadh dehydrogenase subunit m |
| BMEI1112 | 3-oxoacyl-(acyl carrier protein) synthase |
| BMEI1110 | Secretion activator protein |
| BMEI1087 | Beta-hexosaminidase a |
| BMEI1079 | Lipoprotein nlpd |
| BMEI1072 | Hypothetical protein |
| BMEI1050 | Hypothetical protein |
| BMEI1048 | Hypothetical membrane associated protein |
| BMEI1036 | Atp-nad kinase |
| BMEI1034 | Hesb protein |
| BMEI1019 | Molybdenum cofactor biosynthesis protein a |
| BMEI1009 | Hypothetical protein |
| BMEI1007 | 25 kda outer-membrane immunogenic protein precursor |
| BMEI1006 | Hypothetical cytosolic protein |
| BMEI1000 | Hypothetical protein |
| BMEI0989 | Thymidylate kinase |
| BMEI0967 | Phosphate acetyltransferase |
| BMEI0965 | Toluene tolerance protein ttg2b |
| BMEI0952 | Hypothetical protein |
| BMEI0942 | Hypothetical protein |
| BMEI0928 | Acetate coa-transferase alpha subunit |
| BMEI0920 | Mazg protein |
| BMEI0917 | Nitroreductase family |
| BMEI0913 | Penicillin-binding protein 6 (d-alanyl-d-alanine carboxypeptidase fraction c) |
| BMEI0896 | Transcriptional regulatory protein, lysr family |
| BMEI0888 | Peptidyl-prolyl cis-trans isomerase a |
| BMEI0879 | Hypothetical protein |
| BMEI0875 | Atp-dependent protease atp-binding subunit |
| BMEI0870 | Hypothetical protein |
| BMEI0868 | Nitrogen assimilation regulatory protein ntrx |
| BMEI0840 | Lexa repressor |
| BMEI0826 | Ribosome releasing factor |
| BMEI0790 | Alkaline phosphatase |
| BMEI0783 | Protease do |
| BMEI0768 | 50s ribosomal protein l24 |
| BMEI0758 | 50s ribosomal protein l4 |
| BMEI0745 | 50s ribosomal protein l11 |
| BMEI0733 | Non-heme chloroperoxidase |
| BMEI0731 | Cold shock protein |
| BMEI0722 | Hypothetical protein |
| BMEI0714 | Precorrin-8x methylmutase |
| BMEI0709 | 4-hydroxyphenylacetate 3-monooxygenase |
| BMEI0699 | Hypothetical protein |
| BMEI0698 | Transporter |
| BMEI0626 | Transcriptional regulator, gntr family / multiple substrate aminotransferase |
| BMEI0605 | Bicyclomycin resistance protein |
| BMEI0598 | Hypothetical protein |
| BMEI0597 | Serine/threonine protein kinase |
| BMEI0574 | Udp-n-acetylmuramoylalanyl-d-glutamate--2, 6-diaminopimelate ligase |
| BMEI0564 | Dnaj-like protein djla |
| BMEI0559 | 5,10-methylenetetrahydrofolate reductase |
| BMEI0554 | Trans-aconitate methyltransferase |
| BMEI0547 | Phna protein |
| BMEI0524 | Hypothetical protein |
| BMEI0518 | Cold shock protein cspa |
| BMEI0515 | Hypothetical protein |
| BMEI0509 | Lipopolysaccharide core biosynthesis mannosyltransferase lpcc |
| BMEI0506 | Transporter, dme family |
| BMEI0483 | Ribose-phosphate pyrophosphokinase |
| BMEI0458 | Hypothetical membrane spanning protein |
| BMEI0442 | Hypothetical protein |
| BMEI0403 | Multiple antibiotic resistance protein marc |
| BMEI0367 | Hypothetical protein |
| BMEI0355 | Glutamine amidotransferase class-ii |
| BMEI0353 | Proline/betaine transporter |
| BMEI0351 | Adenylosuccinate synthetase |
| BMEI0337 | Biopolymer transport exbd protein |
| BMEI0318 | 2fe-2s ferredoxins, iron-sulfur binding protein |
| BMEI0311 | Transketolase |
| BMEI0301 | Hypothetical protein |
| BMEI0288 | Abc transporter atp-binding protein uup |
| BMEI0280 | Rna polymerase sigma factor |
| BMEI0279 | Transcriptional regulator |
| BMEI0273 | Glcg protein |
| BMEI0261 | High-affinity branched-chain amino acid transport atp-binding protein brag |
| BMEI0209 | Nicotinic acid mononucleotide adenyltransferase |
| BMEI0196 | Protein erfk/srfk |
| BMEI0176 | Porphobilinogen deaminase |
| BMEI0174 | Glycerol-3-phosphate dehydrogenase (nad(p)+) |
| BMEI0146 | Hypothetical protein |
| BMEI0129 | Hydroxyacylglutathione hydrolase, mitochondrial |
| BMEI0126 | Mutator mutt protein (7,8-dihydro-8-oxoguanine-triphosphatase) (8-oxo-dgtpase) |
| BMEI0124 | Bifunctional ornithine acetyltransferase/n-acetylglutamate synthase protein |
| BMEI0116 | Transcriptional regulatory protein, lysr family |
| BMEI0108 | Asparagine transport atp-binding protein |
| BMEI0103 | Methionine gamma-lyase |
| BMEI0099 | 3-hydroxybutyryl-coa dehydrogenase |
| BMEI0098 | Hypothetical protein |
| BMEI0084 | Diaminopimelate decarboxylase |
| BMEI0082 | Hypoxanthine-guanine phosphoribosyltransferase |
| BMEI0075 | 1-acyl-sn-glycerol-3-phosphate acyltransferase |
| BMEI0065 | Hypothetical cytosolic protein |
| BMEI0034 | N-acyl-l-amino acid amidohydrolase |
| BMEI0023 | 3-hydroxybutyryl-coa dehydratase |
| BMEI0010 | Chromosome partitioning protein parb |
